# Adaptive Event-Triggered Synchronization of Uncertain Fractional Order Neural Networks with Double Deception Attacks and Time-Varying Delay

**DOI:** 10.3390/e23101291

**Published:** 2021-09-30

**Authors:** Zhuan Shen, Fan Yang, Jing Chen, Jingxiang Zhang, Aihua Hu, Manfeng Hu

**Affiliations:** School of Science, Jiangnan University, Wuxi 214122, China; 6191204005@stu.jiangnan.edu.cn (Z.S.); 6191204018@stu.jiangnan.edu.cn (F.Y.); 8201703038@jiangnan.edu.cn (J.C.); zhangjingxiang@jiangnan.edu.cn (J.Z.); aihuahu@jiangnan.edu.cn (A.H.)

**Keywords:** uncertain fractional order neural network, adaptive event-triggered scheme, double deception attacks, time-varying delay

## Abstract

This paper investigates the problem of adaptive event-triggered synchronization for uncertain FNNs subject to double deception attacks and time-varying delay. During network transmission, a practical deception attack phenomenon in FNNs should be considered; that is, we investigated the situation in which the attack occurs via both communication channels, from S-C and from C-A simultaneously, rather than considering only one, as in many papers; and the double attacks are described by high-level Markov processes rather than simple random variables. To further reduce network load, an advanced AETS with an adaptive threshold coefficient was first used in FNNs to deal with deception attacks. Moreover, given the engineering background, uncertain parameters and time-varying delay were also considered, and a feedback control scheme was adopted. Based on the above, a unique closed-loop synchronization error system was constructed. Sufficient conditions that guarantee the stability of the closed-loop system are ensured by the Lyapunov-Krasovskii functional method. Finally, a numerical example is presented to verify the effectiveness of the proposed method.

## 1. Introduction

Neural networks, which bridge the micro-world of communications with the physical world for processing information as mathematical models, widely exist in a broad range of areas, such as intelligent control, secure communication, and pattern recognition [1,2,3,4]. Due to the complexity of the dynamic characteristics of some physical systems, a traditional integer-order neural network model cannot accurately represent their dynamic behaviors. Fractional order calculus is not only a generalized form of the traditional integer-order calculus; it also has some irreplaceable properties of integral order calculus, such as the special feature of time memory [4,5,6,7]. Based on these features, the fractional order differential equation has been used to model neural networks [8,9,10,11,12]. Synchronization, among several phenomena arising from the complex nonlinear dynamics of neural networks, has gained lots of attention and has been applied in many integer-order neural networks [13,14,15,16,17]. However, there are few studies about the synchronization problem of FNNs, which was the first motivation of this paper.

The event-triggered scheme (ETS) depends on a predefined event-triggered condition to determine whether the sampled data should be transmitted to the next control unit rather than a fixed period; therefore, replacing the time-triggered scheme (TTS) to save network communication resources and guarantee the system’s performance simultaneously was suggested in [16,18,19,20,21,22,23]. Although ETS was adopted in the latest three studies of different fractional order, real-valued systems [21,22,23], there was still a common disadvantage: the threshold coefficients of traditional ETS are all constants and cannot be timely adjusted to fit a system’s evolution. However, the adaptive event-triggered scheme (AETS), as a combination of adaptive control and traditional ETS, can overcome the conservativeness to make good use of communication resources dynamically. Therefore, designing an AETS with an adaptive threshold coefficient for FNNs to further improve the utilization of communication resources was the second motivation of the current work.

On the other hand, a security problem, due to advanced modern communication technology, has recently emerged as a hot topic in the engineering applications [24,25], especially in autonomous vehicle platooning [26,27]. Since the control components such as sensors, controllers, and actuators are connected by the shared communication networks to achieve remote control, compromise by malicious adversaries is extremely risky [22,28,29]. As a typical representative of malicious attacks, a deception attack can replace the original data with false data to destroy the system [22,28,29,30,31]. To the best of the authors’ knowledge, the synchronization problem of FNNs regarding deception attacks has been investigated in the literature [22], although the deception attacks were only allowed to occur in the controller to actuator (C-A) channel, governed by a Bernoulli variable. However, in communication networks, attacks may occur in the sensor to controller (S-C) channel and C-A channel simultaneously. Moreover, it is well known that a Bernoulli process is a special kind of the Markov process. Therefore, inspired by the aforementioned discussion, investigating double deception attacks governed by Markov processes in the synchronization of FNNs under AETS was the third motivation. Given the actual environmental conditions, neural networks inevitably suffer from noise and limitations of equipment, so uncertainties in parameters and time-varying delay have also been taken into account. The main contributions are outlined below.

(1) The synchronization problem of FNNs under network attacks is firstly proposed with an AETS to further save network bandwidth resources. The AETS has an adaptive law for adjusting its threshold coefficient such that the controller can timely access system information to stabilize the error system.
(2) A generalized deception attack for FNNs is investigated; that is, the deception attack may occur in S-C and C-A channels simultaneously. Moreover, the attack behaviors are governed by independent Markov processes that are more extensive than the Bernoulli processes in other studies.
(3) Parameters’ uncertainties and time-varying delay are also investigated in light of the synchronization problem of FNNs and a double deception attack in the AETS. That is more practicable to some extent.

The remainder of this paper is organized as follows. In Section 2, some preliminaries are introduced and the model is formulated. The main results, including theorems, are shown in Section 3. In Section 4, a simulation which verified the main results is presented. Finally, the discussion and conclusions are presented in Section 5.

Notation: In this paper, $R^{n}$ and ∥·∥ denote the *n*-dimensional Euclidean vector space and the Euclidean norm for vectors, respectively. $R^{n\times n}$ is the set of all n×n real matrices. *T* denotes the transposition of the vectors or matrices. *I* represents the identity matrix with appropriate dimensions, and $He\left[A\right]=A+A^{T}$. The symbol *N* represents the sets of all natural numbers and $N_{0}=N\cup \left\{0\right\}$. The signal “*” denotes the symmetric block of matrix. col(…) and diag(…) represent a column vector and a diagonal matrix, respectively.

**Figure 1:**
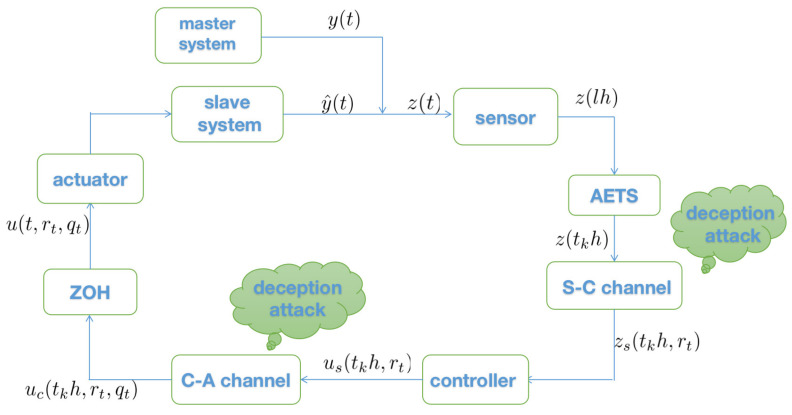
The framework of the closed-loop synchronization error system.

**Remark** **1.** *Network attacks may occur in both S-C and C-A channels during network transmission, as shown in Figure 1. We only found a few studies investigating relevant network attacks, and they only used single-channel attacks: the C-A channel [22]; the S-C channel [32,33,34]. In addition, in prior studies the behaviors of network attacks were governed by Bernoulli variables, usually. To the authors’ knowledge, there is no literature simultaneously considering network attacks in S-C and C-A channels in FNNs. Moreover, in this paper, the double network attacks governed by two independent Markov processes are more general than Bernoulli processes.*

## 2. Preliminaries and Model Formulation

In this section, the basic definitions and relations about fractional calculus are introduced; then a closed-loop synchronization error system is constructed.

### 2.1. Fractional Order Calculations

**Definition** **1.** *The fractional integral of order r for an integrable function $f\left(x\right):\left[t_{0},+\infty \right]\to R$ is defined as [19]:*

$${}_{t_{0}}I_{t}^{r}f\left(t\right)=\frac{1}{\Gamma (r)}\int_{t_{0}}^{t}\frac{f(\beta )}{{\left(t-\beta \right)}^{1-r}}\;d\beta ,$$

*where 0<r<1, and Γ(·) is the Gamma function.*

**Definition** **2.** *The Caputo fractional derivative of order r>0 for a function $f\left(t\right)\in C^{n}\left(\left[t_{0},+\infty \right),R\right)$ is defined as [22]:*

$${}_{t_{0}}D_{t}^{r}f\left(t\right)=\frac{1}{\Gamma (n-r)}\int_{t_{0}}^{t}\frac{f^{\left(n\right)}\left(\beta \right)}{{\left(t-\beta \right)}^{r-n+1}}\;d\beta ,$$

*where $t\geq t_{0}$ and n is an integer such that 0<n−1<r<n. Moreover, when 0<r<1,*

$${}_{t_{0}}D_{t}^{r}f\left(t\right)=\frac{1}{\Gamma (1-r)}\int_{t_{0}}^{t}\frac{f^{{}^{\prime }}\left(\beta \right)}{{\left(t-\beta \right)}^{r}}\;d\beta .$$

From the ldefinitions 1 and 2, it is clear that the Caputo fractional derivative satisfies the following properties:

(1) ${}_{t_{0}}D_{t}^{r}$${}_{t_{0}}I_{t}^{s}f\left(t\right){=}_{t_{0}}D_{t}^{r}$${}_{t_{0}}D_{t}^{-s}f\left(t\right){=}_{t_{0}}D_{t}^{r-s}f\left(t\right)$, where r≥s≥0.
(2) ${}_{t_{0}}D_{t}^{r}C=0$, where *C* is a constant.
(3) ${}_{t_{0}}D_{t}^{r}\left(v_{1}f\left(t\right)+v_{2}g\left(t\right)\right)=v_{1}{}_{t_{0}}D_{t}^{r}f\left(t\right)+v_{2}{}_{t_{0}}D_{t}^{r}g\left(t\right)$, where $v_{1}$ and $v_{2}$ are any constants.

**Lemma** **1** ([22])**.** *For a differentiable function vector $x\left(t\right)\in R^{n}$, an equality with the following form is true:*

$${}_{t_{0}}D_{t}^{r}\left(x^{T}\left(t\right)Px\left(t\right)\right)\leq 2x^{T}\left(t\right)P{}_{t_{0}}D_{t}^{r}x\left(t\right),$$

*where r and $P\in R^{n\times n}$ satisfy 0<r<1 and P>0, respectively.*

**Lemma** **2** ([35])**.** *For a given positive definite matrix $R\in R^{n\times n}$, given scalars a,b satisfying a<b, the following inequality holds for any continuously differentiable function e(x) in $\left[a,b\right]\to R^{n}$:*

$$\left(b-a\right)\int_{a}^{b}e^{T}\left(s\right)Re\left(s\right)\;ds\geq (\int_{a}^{b}e\left(s\right)\;ds{)}^{T}R(\int_{a}^{b}e\left(s\right)\;ds).$$

**Lemma** **3** ([36])**.** For η(t)∈[0,η] and any matrices $R,S\in R^{n\times n}$ satisfying $\left[\begin{array}{cc} R & S\\ {}* & R \end{array}\right]\geq 0$, the following inequality holds:

$$\begin{array}{cc} -\eta \int_{t-\eta }^{t}{\dot{e}}^{T}\left(s\right)R\dot{e}\left(s\right)\;ds & \leq \xi^{T}\left(t\right)\Theta \xi \left(t\right), \end{array}$$

*where ξ(t)=col{e(t),e(t−η(t)),e(t−η)} and*

$$\Theta =\left[\begin{array}{ccc} -R & R-S & S\\ {}* & -2R+He[S] & R-S\\ {}* & * & -R \end{array}\right].$$

**Lemma** **4** ([32])**.** *For given matrix $S=\left[\begin{array}{cc} S_{11} & S_{12}\\ S_{21} & S_{22} \end{array}\right]$, where $S_{12}=S_{21}^{T}$, the following conditions are equivalent.*

$$\begin{array}{cc} \left(1\right) & S<0;\\ \left(2\right) & S_{22}<0,S_{11}-S_{21}S_{22}^{-1}S_{12}<0. \end{array}$$

### 2.2. Model Formulation

Consider the following uncertain FNN model as the master system:

(1) $$\begin{array}{cc} {}_{t_{0}}D_{t}^{r}x\left(t\right) & =-\left(A+\Delta A(t)\right)x\left(t\right)+\left(B+\Delta B(t)\right)\hat{f}\left(x\left(t\right)\right)\\ & \;\;\;\;\;\;\;+\left(D+\Delta D(t)\right)\hat{f}\left(x\left(t-\eta \left(t\right)\right)\right)+I\left(t\right),\\ y(t) & =Cx(t),\\ x(t_{0}) & =\phi_{1}\left(t_{0}\right),t_{0}\in \left[-\eta ,0\right], \end{array}$$

where 0<r<1 denotes the order of fractional order derivative. $x\left(t\right)=(x_{1}\left(t\right),x_{2}\left(t\right),\ldots$, $x_{n}{\left(t\right))}^{T}\in R^{n}$ is the state vector of the neuron. y(t) is the measurable output vector. η(t) satisfies 0≤η(t)≤η, and $\dot{\eta }\left(t\right)\leq \bar{\eta }$ denotes the time-varying coupling delay. $\hat{f}\left(x\left(t\right)\right)=({\hat{f}}_{1}\left(x_{1}\left(t\right)\right),{\hat{f}}_{2}\left(x_{2}\left(t\right)\right),\ldots$, ${\hat{f}}_{n}\left(x_{n}\left(t\right)\right))$ and $\hat{f}\left(x\left(t-\eta \left(t\right)\right)\right)=\left({\hat{f}}_{1}\left(x_{1}\left(t-\eta \left(t\right)\right)\right),{\hat{f}}_{2}\left(x\left(t-\eta \left(t\right)\right)\right),\ldots ,{\hat{f}}_{n}\left(x\left(t-\eta \left(t\right)\right)\right)\right)\in R^{n}$ are the activation functions. I(t) is an external input vector. $A=diag\left(a_{1},a_{2},\ldots ,a_{n}\right)\in R^{n\times n},$ are the self-feedback connection weight matrices. $B={\left(b_{ij}\right)}_{n\times n}\in R^{n\times n},D={\left(d_{ij}\right)}_{n\times n}\in R^{n\times n}$ are the connection weight matrices. Furthermore, ΔA(t),ΔB(t),ΔD(t) are the matrices with time-varying parameters, which are norm bounded and satisfy

$$\left[\Delta A(t),\Delta B(t),\Delta D(t)\right]=GS\left(t\right)\left[E_{a},E_{b},E_{d}\right],$$

where $G,E_{a},E_{b},E_{d}$ are known constant matrices, S(t) is an unknown time-varying matrix function satisfying $S^{T}\left(t\right)S\left(t\right)\leq I$. Assume that master system (1) have a unique solution with initial value $\phi_{1}\left(t_{0}\right)$ and that it is continuously differential on $t_{0}\in \left[-\eta ,0\right]$ [37].

Next, consider the corresponding slave system as follows:

(2) $$\begin{array}{cc} {}_{t_{0}}D_{t}^{r}\hat{x}\left(t\right) & =-\left(A+\Delta A(t)\right)\hat{x}\left(t\right)+\left(B+\Delta B(t)\right)\hat{f}\left(\hat{x}\left(t\right)\right)\\ & \;\;\;\;\;\;\;+\left(D+\Delta D(t)\right)\hat{f}\left(\hat{x}\left(t-\eta \left(t\right)\right)\right)+I\left(t\right)+u\left(t\right),\\ \hat{y}\left(t\right) & =C\hat{x}\left(t\right),\\ \hat{x}\left(t_{0}\right) & =\phi_{2}\left(t_{0}\right),t_{0}\in \left[-\eta ,0\right], \end{array}$$

where $\hat{x}\left(t\right)={\left({\hat{x}}_{1}\left(t\right),y_{2}\left(t\right),\ldots ,{\hat{x}}_{n}\left(t\right)\right)}^{T}$ is the state vector. Similarly, assume slave system (2) also has a unique solution with initial value $\phi_{2}\left(t_{0}\right)$, which is continuously differential on $t_{0}\in \left[-\eta ,0\right]$, and u(t) is the control input, and the others are same as the master system.

In order to realize the synchronization between systems (1) and (2), define the synchronization error $z\left(t\right)=C(\hat{x}\left(t\right)-x\left(t\right))$, and the parameter uncertainty of each part is treated as a whole. The following error system can be obtained:

(3) $$\begin{array}{cc} {}_{t_{0}}D_{t}^{r}e\left(t\right) & =-Ae(t)+Bf(e(t))+Df(e(t-\eta (t)))+Gm(t)+u(t),\\ m(t) & =S\left(t\right)(-E_{a}e\left(t\right)+E_{b}f\left(e\left(t\right)\right)+E_{d}f\left(e\left(t-\eta \left(t\right)\right)\right)),\\ z(t) & =Ce(t),\\ e(t_{0}) & =\phi \left(t_{0}\right),t_{0}\in \left[-\eta ,0\right], \end{array}$$

where $f\left(e\left(t\right)\right)=\hat{f}\left(\hat{x}\left(t\right)\right)-\hat{f}\left(x\left(t\right)\right),f\left(e\left(t-\eta \left(t\right)\right)\right)=\hat{f}\left(\hat{x}\left(t-\eta \left(t\right)\right)\right)-\hat{f}\left(x\left(t-\eta \left(t\right)\right)\right)$. The initial value of error system (3) is $\phi \left(t_{0}\right)=\phi_{2}\left(t_{0}\right)-\phi_{1}\left(t_{0}\right),t_{0}\in \left[-\eta ,0\right]$. It is well known that system (3) has a unique solution [38].

**Remark** **2.** *The model considered in this paper can be regarded as a generalization of [22]. Such an attack has only been considered in the C-A channel and governed by a Bernoulli process in FNNs [22], in which the event-triggered threshold coefficient is a constant and cannot fit a system’s evolution dynamically. The FNNs studied in this paper not only adopt AETS to further improve the utilization of communication resources, but parameters’ uncertainties and double deception attacks are also investigated.*

The following assumption will be used later on.

**Assumption** **1.** *The neuron activation function f(e(t)) is continuous and bounded, and satisfies the following conditions:*

(4) $$\begin{array}{c} 0\leq \frac{f_{i}\left(e_{1}\left(t\right)\right)-f_{i}\left(e_{2}\left(t\right)\right)}{e_{1}\left(t\right)-e_{2}\left(t\right)}\leq \phi_{i}, \end{array}$$

*for i=1,2,…,n, where $\phi_{i}$ are known positive constants.*

Let the two adversary network attacks during the communication be characterized by two independent right-continuous Markov processes $r_{t},q_{t}$ on the probability space taking values in the finite state space M={1,2,…,s} with generator $\pi ={\left(\pi_{ij}\right)}_{s\times s},\rho ={\left(\rho_{ij}\right)}_{s\times s}$ given by

$$Pr\left\{r_{t+k}=j|r_{t}=i\right\}=\left\{\begin{array}{cc} \pi_{ij}k+o\left(k\right) & i\neq j,\\ 1+\pi_{ii}k+o\left(k\right) & i=j. \end{array}\right.$$

$$Pr\left\{q_{t+k}=n|q_{t}=m\right\}=\left\{\begin{array}{cc} \rho_{mn}k+o\left(k\right) & m\neq n,\\ 1+\rho_{mn}k+o\left(k\right) & m=n. \end{array}\right.$$

where $k>0,\lim_{k\to 0}\frac{o(k)}{k}=0,\pi_{ij}\geq 0,i\neq j,\rho_{mn}\geq 0,m\neq n,$ and for every $i,m\in M,\pi_{ii}=-\sum_{j\neq i}\pi_{ij},\rho_{mm}=-\sum_{n\neq m}\rho_{mn}.$

To save on network bandwidth as much as possible, an AETS was adopted in this study. The sensor with sampling period *h* was time-driven, and the output error z(t) was measured by the sensor at the sampling instant $lh,l\in N_{0}$. Let $t_{k}h$ denote the triggered instant; then the next triggered instant is denoted by $t_{k+1}h$. $t_{k}+ih,i\in N$ denotes the current sampling time. Whether or not the sampled data $z(t_{k}+ih)$ should be transmitted is determined by the adaptive event-triggered condition:

(5) $$\begin{array}{c} {\tilde{z}}_{k}^{T}\left(t\right)\Omega {\tilde{z}}_{k}\left(t\right)-d\left(t\right)z^{T}\left(t_{k}+ih\right)\Omega z\left(t_{k}+ih\right)\leq 0, \end{array}$$

where ${\tilde{z}}_{k}\left(t\right)=z\left(t_{k}h\right)-z\left(t_{k}+ih\right),z\left(t_{k}h\right)$ denotes the latest transmitted data, Ω>0 is a weighting matrix to be designed, and the adaptive threshold coefficient d(t) satisfies the following adaptive law:

(6) $$\begin{array}{c} \dot{d}\left(t\right)=\left(\frac{1}{d{\left(t\right)}^{2}}-\frac{\bar{w}}{d(t)}\right){\tilde{z}}_{k}^{T}\left(t\right)\Omega {\tilde{z}}_{k}\left(t\right), \end{array}$$

where $\bar{w}\geq 1$ can adjust the monotonicity of d(t) [32], and the next triggered instant can be denoted as follows:

$$t_{k+1}h=t_{k}h+min\left\{ih|{\tilde{z}}_{k}^{T}\Omega {\tilde{z}}_{k}>d\left(t\right)z^{T}\left(t_{k}+ih\right)\Omega z\left(t_{k}+ih\right),i\in N\right\}.$$

Based on the reality of the network communication, the delay $s_{k}$ is considered at the instant $t_{k}h$. Assume that $0\leq s_{k}\leq \bar{s}$, where $\bar{s}=max\left\{s_{k}\right\}$. The sampling date $z(t_{k}h)$ will be transmitted at the instant $t_{k}+s_{k}$. Then the time interval $\left[t_{k}h+s_{k},t_{k+1}h+s_{k+1}\right)$ can be divided $I_{0}=\left[t_{k}h+s_{k},t_{k}h+h+\bar{s}\right),I_{i}=\left[t_{k}h+ih+\bar{s},t_{k}h+ih+h+\bar{s}\right),i=1,2,\ldots ,\delta -1$, and $\delta =t_{k+1}-t_{k}-1$, $I_{\delta }=\left[t_{k}h+\delta h+\bar{s},t_{k+1}+d_{k+1}\right)$. Then ${\tilde{z}}_{k}\left(t\right)=z\left(t_{k}h\right)-z\left(t_{k}h+ih\right)$ is equivalent to:

(7) $$\begin{array}{c} {\tilde{z}}_{k}\left(t\right)=\left\{\begin{array}{cc} z\left(t_{k}h\right)-z\left(t_{k}h\right), & t\in I_{0},\\ z\left(t_{k}h\right)-z\left(t_{k}h+ih\right), & t\in I_{i},\\ z\left(t_{k}h\right)-z\left(t_{k}h+\delta h\right), & t\in I_{\delta } \end{array}\right. \end{array}$$

which can be written as

(8) $$\begin{array}{c} {\tilde{z}}_{k}\left(t\right)=z\left(t_{k}h\right)-z\left(t-\tau \left(t\right)\right),t\in \left[t_{k}h+s_{k},t_{k+1}h+s_{k+1}\right) \end{array}$$

in which

(9) $$\begin{array}{c} \tau \left(t\right)=\left\{\begin{array}{cc} t-t_{k}h, & t\in I_{0},\\ t-t_{k}h-ih, & t\in I_{i},\\ t-t_{k}h-\delta h, & t\in I_{\delta }. \end{array}\right. \end{array}$$

According to Equation (9), it is easy to get

$$\begin{array}{c} 0\leq \tau \left(t\right)\leq h+\bar{s},t\in \left[t_{k}h+s_{k},t_{k+1}h+s_{k+1}\right). \end{array}$$

**Remark** **3.** *From the adaptive event-triggered condition (5), it is easy to know the minimum event-triggered interval is a constant, which means that there is no Zeno behavior.*

As shown in Figure 1, deception attacks may occur on the S-C communication channel, and the integrity of normal transmission data will be damaged by malicious attacks. To depict the stochastic occurrence modeling of deception attacks, Markov processes are adopted in this paper. Then the control input in time interval $[t_{k}h+s_{k},t_{k+1}h+s_{k+1}),k=1,2,\ldots ,$ can be denoted as

(10) $$\begin{array}{cc} z_{s}\left(t_{k}h,r_{t}\right) & =b^{s}\left(r_{t}\right)z\left(t_{k}h\right)+{\bar{b}}^{s}\left(r_{t}\right)g_{s}\left(z\left(t_{k}h\right)\right),\\ & =b^{s}\left(r_{t}\right)\left(z\left(t-\tau \left(t\right)\right)+{\tilde{z}}_{k}\left(t\right)\right)+{\bar{b}}^{s}\left(r_{t}\right)g_{s}\left(z\left(t_{k}h\right)\right). \end{array}$$

where $b^{s}\left(1\right)=1,b^{s}\left(2\right)=0,{\bar{b}}^{s}\left(r_{t}\right)=1-b^{s}\left(r_{t}\right),$ and $g_{s}:R^{n}\to R^{n}$ is the energy bounded deception signal in the S-C communication channel satisfying

(11) $$\begin{array}{c} ∥g_{s}\left(x\left(t\right)\right)∥\leq ∥G_{s}x\left(t\right)∥. \end{array}$$

where $G_{s}\in R^{n\times n}$ is a known constant matrix satisfying $G_{s}>0.$ If $r_{t}=1$, the data will be transmitted normally without any attack. Conversely, $r_{t}=2$ means that malicious attack signals occur in the S-C channel.

The main purpose of this study was to synchronize uncertain FNNs under AETS, subject to double deception attacks and time-varying delay. Construct the state feedback controller:

(12) $$\begin{array}{cc} u(t) & =u_{s}\left(t_{k}h,r_{t}\right),\\ & =Kz_{s}\left(t_{k}h,r_{t}\right),t\in \left[t_{k}h+s_{k},t_{k+1}h+s_{k+1}\right), \end{array}$$

where the feedback gain matrix *K* needs to be determined.

In a similar routine to that of the S-C communication channel, when the released data $u_{s}\left(t_{k}h,r_{t}\right)$ are transmitted through the C-A communication channel, the channel may be attacked again. Therefore, the control output signal can be denoted as

(13) $$\begin{array}{cc} u(t) & =u_{c}\left(t_{k}h,r_{t},q_{t}\right),\\ & =b^{c}\left(q_{t}\right)u_{s}\left(t_{k}h,r_{t}\right)+{\bar{b}}^{c}\left(q_{t}\right)g_{c}\left(u_{s}\left(t_{k}h,r_{t}\right)\right),\\ & =b^{c}\left(q_{t}\right)b^{s}\left(r_{t}\right)KCe\left(t-\tau \left(t\right)\right)+b^{c}\left(q_{t}\right)b^{s}\left(r_{t}\right)K\tilde{z}\left(t\right)+b^{c}\left(q_{t}\right){\bar{b}}^{s}\left(r_{t}\right)Kg_{s}\left(\bar{z}\left(t\right)\right)\\ & \;\;\;\;\;+{\bar{b}}^{c}\left(q_{t}\right)g_{c}\left(u_{s}\left(t_{k}h,r_{t}\right)\right),\;\;\;\;\;\;\;t\in \left[t_{k}h+s_{k},t_{k+1}h+s_{k+1}\right), \end{array}$$

where $\bar{z}\left(t\right)=\tilde{z}\left(t\right)+z\left(t-\tau \left(t\right)\right)$, $b^{c}\left(1\right)=1,b^{c}\left(2\right)=0,{\bar{b}}^{c}\left(q_{t}\right)=1-b^{c}\left(q_{t}\right),$ and $g_{c}:R^{n}\to R^{n}$ is the energy bounded deception signal in the C-A communication channel satisfying

(14) $$\begin{array}{c} ∥g_{c}\left(x\left(t\right)\right)∥\leq ∥G_{c}x\left(t\right)∥. \end{array}$$

where $G_{c}\in R^{n\times n}$ is a known constant matrix satisfying $G_{c}>0$. For simplicity, for every $i,m\in M,r_{t}=i,q_{t}=m$, $b^{s}\left(r_{t}\right),b^{c}\left(q_{t}\right)$ are denoted in this paper by $b_{i}^{s}$ and $b_{m}^{c}$, respectively. Similarly, for a matrix $P_{1}\left(r_{t},q_{t}\right)$, it is denoted by $P_{1}^{im}$. In addition, for a matrix $P_{1}^{im}$, there is the following definition:

(15) $$\begin{array}{c} {\bar{P}}_{1}^{im}=\sum_{j\in M}\pi_{ij}P_{1}^{jm}+\sum_{n\in M}\rho_{mn}P_{1}^{in}. \end{array}$$

Then, it is easy to obtain the error system

(16) $$\begin{array}{cc} {}_{t_{0}}D_{t}^{r}e\left(t\right) & =-Ae\left(t\right)+Bf\left(e\left(t\right)\right)+Df\left(e\left(t-\eta \left(t\right)\right)\right)+Gm\left(t\right)+b_{m}^{c}b_{i}^{s}K\tilde{z}\left(t\right)\\ & \;\;\;\;\;+b_{m}^{c}b_{i}^{s}KCe\left(t-\tau \left(t\right)\right)+b_{m}^{c}{\bar{b}}_{i}^{s}Kg_{s}\left(\bar{z}\left(t\right)\right)+{\bar{b}}_{m}^{c}g_{c}\left(u_{s}\left(t_{k}h,r_{t}\right)\right),\\ m(t) & =S\left(t\right)(-E_{a}e\left(t\right)+E_{b}f\left(e\left(t\right)\right)+E_{d}f\left(e\left(t-\eta \left(t\right)\right)\right)),\\ z(t) & =Ce(t),\\ e(t_{0}) & =\phi \left(t_{0}\right),t_{0}\in \left[-max\left\{\eta ,h\right\},0\right]. \end{array}$$

The following two definitions will be used in the proof of Theorem 1.

**Definition** **3** ([39])**.** *Let $V(t,e\left(t\right),r_{t}=i,q_{t}=m)$ be the positive Lyapunov–Krasovskii functional and L(·) be a weak infinitesimal operator. Then*

$$E\left\{\int_{0}^{t}LV\left(s,e\left(s\right),i,m\right)\;ds\right\}=EV\left(t,e\left(t\right),i,m\right)-EV\left(0,\phi \left(t_{0}\right),r_{0},q_{0}\right),$$

*where E denotes the expectation.*

**Definition** **4** ([40,41])**.** *The synchronization error system (16) is said to be globally, stochastically, asymptotically stable in the mean square sense, if for any initial conditions $\phi (t_{0})$ defined on [−max{η,h},0] and $r_{0},q_{0}\in M$ the following condition is satisfied:*

$$\lim_{t\to \infty }E\left\{\int_{0}^{t}e^{T}\left(s\right)e\left(s\right)\;ds|\phi \left(t_{0}\right),r_{0},q_{0}\right\}<\infty .$$

So far, a closed-loop synchronization error system (16) has been constructed. In the following, in order to realize the synchronization between systems (1) and (2), the stability of error system (16) will be proven.

## 3. Results

Two theorems are developed in this section. Firstly, the synchronization criterion for systems (1) and (2) is presented in Theorem 1. Then, on the basis of Theorem 1, the criterion for feedback controller design is developed by Theorem 2.

**Theorem** **1.** *Suppose Assumption 1 holds. The FNNs (1) and (2) are globally, stochastically, asymptotically synchronized under the feedback control scheme (12) in the mean square sense, for the given scalars r and control gain matrix K, if there exist positive definite matrices $P,\Omega ,P_{1}^{im},P_{3}^{im},N_{1}$, $N_{3},R_{1}^{im},R_{2}^{im}$, $M_{1}^{im},M_{2}^{im},L_{1},L_{2},J_{1},J_{2},Q_{1},Q_{2}$; positive definite diagonal matrices $\Delta_{1}$, $\Delta_{2}$; and matrices $P_{2}^{im},N_{2}$, $S^{im},T^{im}$; and positive scalars $\varepsilon ,\lambda_{1},\lambda_{2},$ such that the following LMIs for every i,m hold:*

(17) $$\begin{array}{c} \left[\begin{array}{ccccccc} \Pi_{1,1} & \Pi_{1,2} & \Pi_{1,3} & \Pi_{1,4} & \Pi_{1,5} & \Pi_{1,6} & \Pi_{1,7}\\ {}* & \Pi_{2,2} & \Pi_{2,3} & 0 & \Pi_{2,5} & 0 & 0\\ {}* & * & \Pi_{3,3} & 0 & 0 & 0 & \Pi_{3,7}\\ {}* & * & * & \Pi_{4,4} & 0 & \Pi_{4,6} & \Pi_{4,7}\\ {}* & * & * & * & \Pi_{5,5} & 0 & \Pi_{5,7}\\ {}* & * & * & * & * & \Pi_{6,6} & 0\\ {}* & * & * & * & * & * & \Pi_{7,7} \end{array}\right]<0, \end{array}$$

(18) $$\begin{array}{c} {\bar{R}}_{1}^{im}<L_{1},{\bar{R}}_{2}^{im}<L_{2},{\bar{M}}_{1}^{im}<J_{1},{\bar{M}}_{2}^{im}<J_{1}, \end{array}$$

(19) $$\begin{array}{c} \left[\begin{array}{cc} R_{2}^{im} & S^{im}\\ {}* & R_{2}^{im} \end{array}\right]\geq 0,\left[\begin{array}{cc} M_{2}^{im} & T^{im}\\ {}* & M_{2}^{im} \end{array}\right]\geq 0, \end{array}$$

(20) $$\begin{array}{c} \left[\begin{array}{cc} P_{1}^{im} & P_{2}^{im}\\ {}* & P_{3}^{im} \end{array}\right]>0,\left[\begin{array}{cc} N_{1} & N_{2}\\ {}* & N_{3} \end{array}\right]>0, \end{array}$$

*where*

$$\begin{array}{cc} \Pi_{1,1} & =\left[\begin{array}{cc} \Xi_{1,1} & \Xi_{1,2}\\ {}* & \Xi_{2,2} \end{array}\right],\Pi_{1,2}=\left[\begin{array}{ccc} S^{im} & M_{2}^{im}-T^{im} & T^{im}\\ R_{2}^{im}-S^{im} & 0 & 0 \end{array}\right],\\ \Pi_{1,3} & =\left[\begin{array}{cc} \Xi_{1,6} & -\varepsilon E_{a}E_{d}+PD\\ 0 & 0 \end{array}\right],\Pi_{6,6}=-{\left(\lambda_{2}G_{c}^{T}G_{c}\right)}^{-1},\\ \Pi_{1,4} & =\left[\begin{array}{ccc} b_{m}^{c}{\bar{b}}_{i}^{s}PK & {\bar{b}}_{m}^{c}P & b_{m}^{c}b_{i}^{s}PK\\ 0 & 0 & \lambda_{1}C^{T}G_{s}^{T}G_{s} \end{array}\right],\Pi_{1,5}=\left[\begin{array}{ccc} P_{1}^{im}+{\left(P_{2}^{im}\right)}^{T} & P_{2}^{im}+P_{3}^{im} & 0\\ 0 & 0 & 0 \end{array}\right],\\ \Pi_{1,6} & =\left[\begin{array}{c} 0\\ b_{i}^{s}C^{T}K^{T} \end{array}\right],\Pi_{1,7}=\Psi ⊗\left[\begin{array}{c} -A^{T}\\ b_{i}^{s}b_{m}^{c}C^{T}K^{T} \end{array}\right],\Pi_{2,3}=\left[\begin{array}{cc} 0 & 0\\ 0 & \Phi \Delta_{2}-\left(1-\bar{\eta }\right)N_{2}\\ 0 & 0 \end{array}\right], \end{array}$$

$$\begin{array}{cc} \Pi_{2,2} & =\left[\begin{array}{ccc} -Q_{1}-R_{2}^{im} & 0 & 0\\ {}* & \Xi_{4,4} & M_{2}^{im}-T^{im}\\ {}* & * & -Q_{2}-M_{2}^{im} \end{array}\right],\Pi_{2,5}=\left[\begin{array}{ccc} -P_{1}^{im} & -P_{2}^{im} & 0\\ 0 & 0 & 0\\ -{\left(P_{2}^{im}\right)}^{T} & -P_{3}^{im} & 0 \end{array}\right],\\ \Pi_{3,3} & =\left[\begin{array}{cc} \Xi_{6,6} & \varepsilon E_{a}E_{d}\\ {}* & \Xi_{7,7} \end{array}\right],\Pi_{3,7}=\Psi ⊗\left[\begin{array}{c} B^{T}\\ D^{T} \end{array}\right],\Pi_{4,4}=\left[\begin{array}{ccc} -\lambda_{1}I & 0 & 0\\ {}* & -\lambda_{2}I & 0\\ {}* & * & \Xi_{10,10} \end{array}\right],\\ \Pi_{4,6} & =\left[\begin{array}{c} {\bar{b}}_{i}^{s}K^{T}\\ 0\\ b_{i}^{s}K^{T} \end{array}\right],\Pi_{4,7}=\Psi ⊗\left[\begin{array}{c} {\bar{b}}_{i}^{s}b_{m}^{c}K^{T}\\ {\bar{b}}_{m}^{c}\\ b_{i}^{s}b_{m}^{c}K^{T} \end{array}\right],\Pi_{5,5}=\left[\begin{array}{ccc} {\bar{P}}_{1}^{im}-R_{1}^{im} & {\bar{P}}_{2}^{im} & 0\\ {}* & {\bar{P}}_{3}^{im}-M_{1}^{im} & 0\\ {}* & * & -\varepsilon I \end{array}\right],\\ \Pi_{5,7} & =\Psi ⊗\left[\begin{array}{c} 0\\ 0\\ G^{T} \end{array}\right],\Pi_{7,7}=\left[\begin{array}{cccc} -{\left(R_{2}^{im}\right)}^{-1} & 0 & 0 & 0\\ {}* & -{\left(M_{2}^{im}\right)}^{-1} & 0 & 0\\ {}* & * & -2h{\left(L_{2}\right)}^{-1} & 0\\ {}* & * & * & -2\eta {\left(J_{2}\right)}^{-1} \end{array}\right],\\ \Xi_{1,1} & =-2PA+Q_{1}+Q_{2}+N_{1}+h^{2}R_{1}^{im}+\eta^{2}M_{1}^{im}+\frac{h^{3}}{2}L_{1}+\frac{\eta^{3}}{2}J_{1}-R_{2}^{im}-M_{2}^{im}+\varepsilon E_{a}^{2},\\ \Xi_{1,2} & =b_{m}^{c}b_{i}^{s}PKC+R_{2}^{im}-S^{im},\Psi =\left[\begin{array}{cccc} h & \eta & h^{2} & \eta^{2} \end{array}\right],\\ \Xi_{2,2} & =-2R_{2}^{im}+He\left[S^{im}\right]+C^{T}\Omega C+\lambda_{1}C^{T}G_{s}^{T}G_{s}C+\Omega ,\\ \Xi_{4,4} & =-\left(1-\bar{\eta }\right)N_{1}-2M_{2}^{im}+He\left[T^{im}\right],\Xi_{6,6}=N_{3}-2\Delta_{1}+\varepsilon E_{b}^{2},\\ \Xi_{7,7} & =-\left(1-\bar{\eta }\right)N_{3}-2\Delta_{2}+\varepsilon E_{d}^{2},\;\;\;\;\;\Xi_{10,10}=\lambda_{1}G_{s}^{T}G_{s}-\bar{w}\Omega ,\\ \Xi_{1,6} & =PB+N_{2}+\Phi \Delta_{1}-\varepsilon E_{a}E_{b}. \end{array}$$

**Proof.**  Consider the following fractional order Lyapunov–Krasovskii functional:

$$V\left(t,e\left(t\right)\right)=\sum_{k=1}^{9}V_{k}\left(t,e\left(t\right),r_{t},q_{t}\right),$$

where

$$\begin{array}{cc} V_{1}\left(t,e\left(t\right),r_{t},q_{t}\right) & ={\;}_{t0}D_{t}^{r-1}e^{T}\left(t\right)Pe\left(t\right),\\ V_{2}\left(t,e\left(t\right),r_{t},q_{t}\right) & =\frac{1}{2}d^{T}\left(t\right)d\left(t\right),\\ V_{3}\left(t,e\left(t\right),r_{t},q_{t}\right) & ={\left[\begin{array}{c} \int_{t-h}^{t}e\left(s\right)\;ds\\ \int_{t-\eta }^{t}e\left(s\right)\;ds \end{array}\right]}^{T}\left[\begin{array}{cc} P_{1}^{im} & P_{2}^{im}\\ {}* & P_{3}^{im} \end{array}\right]\left[\begin{array}{c} \int_{t-h}^{t}e\left(s\right)\;ds\\ \int_{t-\eta }^{t}e\left(s\right)\;ds \end{array}\right],\\ V_{4}\left(t,e\left(t\right),r_{t},q_{t}\right) & =\int_{t-h}^{t}e^{T}\left(s\right)Q_{1}e\left(s\right)\;ds+\int_{t-\eta }^{t}e^{T}\left(s\right)Q_{2}e\left(s\right)\;ds,\\ V_{5}\left(t,e\left(t\right),r_{t},q_{t}\right) & =\int_{t-\eta (t)}^{t}{\left[\begin{array}{c} e(s)\\ f(e(s)) \end{array}\right]}^{T}\left[\begin{array}{cc} N_{1} & N_{2}\\ {}* & N_{3} \end{array}\right]\left[\begin{array}{c} e(s)\\ f(e(s)) \end{array}\right]\;ds,\\ V_{6}\left(t,e\left(t\right),r_{t},q_{t}\right) & =h\int_{-h}^{0}\;\int_{t+\theta }^{t}{\left[\begin{array}{c} e(s)\\ \dot{e}\left(s\right) \end{array}\right]}^{T}\left[\begin{array}{cc} R_{1}^{im} & 0\\ 0 & R_{2}^{im} \end{array}\right]\left[\begin{array}{c} e(s)\\ \dot{e}\left(s\right) \end{array}\right]\;dsd\theta ,\\ V_{7}\left(t,e\left(t\right),r_{t},q_{t}\right) & =\eta \int_{-\eta }^{0}\;\int_{t+\theta }^{t}{\left[\begin{array}{c} e(s)\\ \dot{e}\left(s\right) \end{array}\right]}^{T}\left[\begin{array}{cc} M_{1}^{im} & 0\\ 0 & M_{2}^{im} \end{array}\right]\left[\begin{array}{c} e(s)\\ \dot{e}\left(s\right) \end{array}\right]\;dsd\theta ,\\ V_{8}\left(t,e\left(t\right),r_{t},q_{t}\right) & =h\int_{-h}^{0}\;\int_{\theta }^{0}\;\int_{t+\beta }^{t}{\left[\begin{array}{c} e(s)\\ \dot{e}\left(s\right) \end{array}\right]}^{T}\left[\begin{array}{cc} L_{1} & 0\\ 0 & L_{2} \end{array}\right]\left[\begin{array}{c} e(s)\\ \dot{e}\left(t\right) \end{array}\right]\;dsd\beta d\theta ,\\ V_{9}\left(t,e\left(t\right),r_{t},q_{t}\right) & =\eta \int_{-\eta }^{0}\;\int_{\theta }^{0}\;\int_{t+\beta }^{t}{\left[\begin{array}{c} e(s)\\ \dot{e}\left(s\right) \end{array}\right]}^{T}\left[\begin{array}{cc} J_{1} & 0\\ 0 & J_{2} \end{array}\right]\left[\begin{array}{c} e(s)\\ \dot{e}\left(t\right) \end{array}\right]\;dsd\beta d\theta . \end{array}$$

For simplicity, $V_{i}=V_{i}\left(t,e\left(t\right),r_{t},q_{t}\right),i=1,2,\ldots ,9$.

The weak infinitesimal operator L is defined as follows:

$$\begin{array}{cc} LV(t,e\left(t\right),r_{t},q_{t}) & =\frac{\partial V(t,e\left(t\right),r_{t},q_{t})}{\partial t}+{\left.{\dot{e}}^{T}\left(t\right)\frac{\partial V(t,e\left(t\right),r_{t},q_{t})}{\partial e(t)}\right|}_{r_{t}=i,q_{t}=m}\\ & +\sum_{j=1}^{2}\pi_{ij}V\left(e\left(t\right),j,m\right)+\sum_{n=1}^{2}\rho_{mn}V\left(e\left(t\right),i,n\right). \end{array}$$

By calculating the weak infinitesimal derivatives of $V(t,e\left(t\right),r_{t},q_{t})$ along with the error system (16), one has

(21) $$\begin{array}{cc} LV_{1} & \leq 2e^{T}\left(t\right)PD_{t}^{\gamma }e\left(t\right),\;\;\;\;\;\;\;LV_{2}=d\left(t\right)\dot{d}\left(t\right), \end{array}$$

(22) $$\begin{array}{cc} LV_{3} & =2{\left[\begin{array}{c} \int_{t-h}^{t}e\left(s\right)\;ds\\ \int_{t-\eta }^{t}e\left(s\right)\;ds \end{array}\right]}^{T}\left[\begin{array}{cc} P_{1}^{im} & P_{2}^{im}\\ {}* & P_{3}^{im} \end{array}\right]\left[\begin{array}{c} e(t)-e(t-h)\\ e(t)-e(t-\eta ) \end{array}\right]+{\left[\begin{array}{c} \int_{t-h}^{t}e\left(s\right)\;ds\\ \int_{t-\eta }^{t}e\left(s\right)\;ds \end{array}\right]}^{T}\\ & \times \left[\begin{array}{cc} {\bar{P}}_{1}^{im} & {\bar{P}}_{2}^{im}\\ {}* & {\bar{P}}_{3}^{im} \end{array}\right]\left[\begin{array}{c} \int_{t-h}^{t}e\left(s\right)\;ds\\ \int_{t-\eta }^{t}e\left(s\right)\;ds \end{array}\right], \end{array}$$

(23) $$\begin{array}{cc} LV_{4} & =e^{T}\left(t\right)\left(Q_{1}+Q_{2}\right)e\left(t\right)-e^{T}\left(t-h\right)Q_{1}e\left(t-h\right)-e^{T}\left(t-\eta \right)Q_{2}e\left(t-\eta \right), \end{array}$$

(24) $$\begin{array}{cc} LV_{5} & ={\left[\begin{array}{c} e(t)\\ f(e(t)) \end{array}\right]}^{T}\left[\begin{array}{cc} N_{1} & N_{2}\\ {}* & N_{3} \end{array}\right]\left[\begin{array}{c} e(t)\\ f(e(t)) \end{array}\right]-\left(1-\bar{\eta }\right){\left[\begin{array}{c} e(t-\eta (t))\\ f(e(t-\eta (t))) \end{array}\right]}^{T}\\ & \times \left[\begin{array}{cc} N_{1} & N_{2}\\ {}* & N_{3} \end{array}\right]\left[\begin{array}{c} e(t-\eta (t))\\ f(e(t-\eta (t))) \end{array}\right], \end{array}$$

(25) $$\begin{array}{cc} LV_{6} & =h^{2}{\left[\begin{array}{c} e(t)\\ \dot{e}\left(t\right) \end{array}\right]}^{T}\left[\begin{array}{cc} R_{1}^{im} & 0\\ 0 & R_{2}^{im} \end{array}\right]\left[\begin{array}{c} e(t)\\ \dot{e}\left(t\right) \end{array}\right]-h\int_{t-h}^{t}\left[\begin{array}{c} e(s)\\ \dot{e}\left(s\right) \end{array}\right]\left[\begin{array}{cc} R_{1}^{im} & 0\\ 0 & R_{2}^{im} \end{array}\right]\left[\begin{array}{c} e(t)\\ \dot{e}\left(t\right) \end{array}\right]ds\\ & +h\int_{-h}^{0}\;\int_{t+\theta }^{t}\left[\begin{array}{c} e(t)\\ \dot{e}\left(t\right) \end{array}\right]\left[\begin{array}{cc} {\bar{R}}_{1}^{im} & 0\\ 0 & {\bar{R}}_{2}^{im} \end{array}\right]\left[\begin{array}{c} e(t)\\ \dot{e}\left(t\right) \end{array}\right]dsd\theta , \end{array}$$

(26) $$\begin{array}{cc} LV_{7} & =\eta^{2}{\left[\begin{array}{c} e(t)\\ \dot{e}\left(t\right) \end{array}\right]}^{T}\left[\begin{array}{cc} M_{1}^{im} & 0\\ 0 & M_{2}^{im} \end{array}\right]\left[\begin{array}{c} e(t)\\ \dot{e}\left(t\right) \end{array}\right]-\eta \int_{t-\eta }^{t}\left[\begin{array}{c} e(s)\\ \dot{e}\left(s\right) \end{array}\right]\left[\begin{array}{cc} M_{1}^{im} & 0\\ 0 & M_{2}^{im} \end{array}\right]\left[\begin{array}{c} e(t)\\ \dot{e}\left(t\right) \end{array}\right]ds\\ & +\eta \int_{-\eta }^{0}\;\int_{t+\theta }^{t}\left[\begin{array}{c} e(t)\\ \dot{e}\left(t\right) \end{array}\right]\left[\begin{array}{cc} {\bar{M}}_{1}^{im} & 0\\ 0 & {\bar{M}}_{2}^{im} \end{array}\right]\left[\begin{array}{c} e(t)\\ \dot{e}\left(t\right) \end{array}\right]dsd\theta , \end{array}$$

(27) $$\begin{array}{cc} LV_{8} & =\frac{h^{3}}{2}{\left[\begin{array}{c} e(t)\\ \dot{e}\left(t\right) \end{array}\right]}^{T}\left[\begin{array}{cc} L_{1} & 0\\ 0 & L_{2} \end{array}\right]\left[\begin{array}{c} e(t)\\ \dot{e}\left(t\right) \end{array}\right]-h\int_{-h}^{0}\;\int_{t+\theta }^{t}{\left[\begin{array}{c} e(t)\\ \dot{e}\left(t\right) \end{array}\right]}^{T}\left[\begin{array}{cc} L_{1} & 0\\ 0 & L_{2} \end{array}\right]\left[\begin{array}{c} e(t)\\ \dot{e}\left(t\right) \end{array}\right]dsd\theta , \end{array}$$

(28) $$\begin{array}{cc} LV_{9} & =\frac{\eta^{3}}{2}{\left[\begin{array}{c} e(t)\\ \dot{e}\left(t\right) \end{array}\right]}^{T}\left[\begin{array}{cc} J_{1} & 0\\ 0 & J_{2} \end{array}\right]\left[\begin{array}{c} e(t)\\ \dot{e}\left(t\right) \end{array}\right]-\eta \int_{-\eta }^{0}\;\int_{t+\theta }^{t}{\left[\begin{array}{c} e(t)\\ \dot{e}\left(t\right) \end{array}\right]}^{T}\left[\begin{array}{cc} J_{1} & 0\\ 0 & J_{2} \end{array}\right]\left[\begin{array}{c} e(t)\\ \dot{e}\left(t\right) \end{array}\right]dsd\theta . \end{array}$$

By using Lemmas 1 and 2, it follows that

(29) $$\begin{array}{cc} -h\int_{t-h}^{t}{\dot{e}}^{T}\left(s\right)R_{2}^{im}\dot{e}\left(s\right)\;ds & \leq \xi_{1}^{T}\left(t\right)\Theta_{1}\xi_{1}\left(t\right), \end{array}$$

(30) $$\begin{array}{cc} -\eta \int_{t-\eta }^{t}{\dot{e}}^{T}\left(s\right)M_{2}^{im}\dot{e}\left(s\right)\;ds & \leq \xi_{2}^{T}\left(t\right)\Theta_{2}\xi_{2}\left(t\right), \end{array}$$

(31) $$\begin{array}{cc} -h\int_{t-h}^{t}e^{T}\left(s\right)R_{1}^{im}e\left(s\right)\;ds & \leq -(\int_{t-h}^{t}e\left(s\right)\;ds{)}^{T}R_{1}^{im}\int_{t-h}^{t}e\left(s\right)\;ds, \end{array}$$

(32) $$\begin{array}{cc} -\eta \int_{t-\eta }^{t}e^{T}\left(s\right)M_{1}^{im}e\left(s\right)\;ds & \leq -(\int_{t-\eta }^{t}e\left(s\right)\;ds{)}^{T}M_{1}^{im}\int_{t-\eta }^{t}e\left(s\right)\;ds, \end{array}$$

where $\xi_{1}\left(t\right)=col\left\{e\left(t\right),e\left(t-\tau \left(t\right)\right),e\left(t-h\right)\right\},\xi_{2}\left(t\right)=col\left\{e\left(t\right),e\left(t-\eta \left(t\right)\right),e\left(t-\eta \right)\right\},$ and

$$\Theta_{1}=\left[\begin{array}{ccc} -R_{2}^{im} & R_{2}^{im}-S^{im} & S^{im}\\ {}* & -2R_{2}^{im}+He\left[S^{im}\right] & R_{2}^{im}-S^{im}\\ {}* & * & -R_{2}^{im} \end{array}\right],$$

$$\Theta_{2}=\left[\begin{array}{ccc} -M_{2}^{im} & M_{2}^{im}-T^{im} & T^{im}\\ {}* & -2M_{2}^{im}+He\left[T^{im}\right] & M_{2}^{im}-T^{im}\\ {}* & * & -M_{2}^{im} \end{array}\right].$$

It can be obtained from m(t) that

(33) $$\begin{array}{c} \begin{array}{c} \varepsilon e^{T}\left(t\right)E_{a}^{2}e\left(t\right)-2\varepsilon e^{T}\left(t\right)E_{a}E_{b}f\left(e\left(t\right)\right)-2\varepsilon e^{T}\left(t\right)E_{a}E_{d}f\left(e\left(t-\eta \left(t\right)\right)\right)\\ +\varepsilon f^{T}\left(e\left(t\right)\right)E_{b}^{2}f\left(e\left(t\right)\right)+2\varepsilon f^{T}\left(e\left(t\right)\right)E_{b}E_{d}f\left(e\left(t-\eta \left(t\right)\right)\right)\\ +\varepsilon f^{T}\left(e\left(t-\eta \left(t\right)\right)\right)E_{d}^{2}f\left(e\left(t-\eta \left(t\right)\right)\right)-\varepsilon m^{T}\left(t\right)m\left(t\right)\geq 0. \end{array} \end{array}$$

Moreover, from the adaptive event-triggered condition, activation function, (11) and (14), it follows that

(34) $$\begin{array}{cc} d\left(t\right)\dot{d}\left(t\right)\leq z^{T}\left(T-\tau \left(t\right)\right) & \Omega z\left(T-\tau \left(t\right)\right)-\bar{w}{\tilde{z}}^{T}\left(t\right)\Omega \tilde{z}\left(t\right), \end{array}$$

(35) $$\begin{array}{cc} -2f^{T}\left(e\left(t\right)\right)\Delta_{1}f\left(e\left(t\right)\right) & +2e^{T}\left(t\right)\Phi \Delta_{1}f\left(e\left(t\right)\right)\geq 0, \end{array}$$

(36) $$\begin{array}{cc} -2f^{T}\left(e\left(t-\eta \left(t\right)\right)\right)\Delta_{2}f\left(e\left(t-\eta \left(t\right)\right)\right) & +2e^{T}\left(t-\eta \left(t\right)\right)\Phi \Delta_{2}f\left(e\left(t-\eta \left(t\right)\right)\right)\geq 0, \end{array}$$

(37) $$\begin{array}{cc} \lambda_{1}{\bar{z}}^{T}\left(t\right)G_{s}^{T}G_{s}\bar{z}\left(t\right) & -\lambda_{1}g_{s}^{T}\left(\bar{z}\left(t\right)\right)g_{s}\left(\bar{z}\left(t\right)\right)\geq 0, \end{array}$$

(38) $$\begin{array}{cc} \lambda_{2}u_{c}^{T}G_{c}^{T}G_{c}u_{c} & -\lambda_{2}g_{c}^{T}\left(u_{c}\right)g_{c}\left(u_{c}\right)\geq 0. \end{array}$$

Let

$$\begin{array}{c} \zeta (t)=col\{e(t),e(t-\tau (t)),e(t-h),e(t-\eta (t)),e(t-\eta ),f(e(t)),f(e(t-\eta (t))),\\ g_{s}\left(\bar{z}\right),g_{c}\left(u_{c}\right),\tilde{z}\left(t\right),\int_{t-h}^{t}e^{T}\left(s\right)\;ds,\int_{t-\eta }^{t}e^{T}\left(s\right)\;ds,m\left(t\right)\}, \end{array}$$

together with (21)–(38). Then, the following can be obtained.

$$LV\left(t,e\left(t\right),r_{t},q_{t}\right)\leq \zeta^{T}\left(t\right)\Xi \zeta \left(t\right).$$

From the aforementioned part, we know that matrix inequality (17) guarantees Ξ<0 holds. That further guarantees that $LV(t,e\left(t\right),r_{t},q_{t})<0$ holds for every i,m∈M.

Let $\lambda_{0}=\lambda_{min}\left(-\Xi \right)$; then $\lambda_{0}>0$. For any t>0, we have:

$$LV\left(t,e\left(t\right),r_{t},q_{t}\right)\leq -\lambda_{0}\zeta^{T}\left(t\right)\zeta \left(t\right)\leq -\lambda_{0}e^{T}\left(t\right)e\left(t\right).$$

By Definition 3, one can obtain:

$$EV\left(t,e\left(t\right),i,m\right)-EV\left(0,\phi \left(t_{0}\right),r_{0},q_{0}\right)\leq -\lambda_{0}E\left\{\int_{0}^{t}e^{T}\left(t\right)e\left(t\right)\;ds\right\},$$

hence, for t≥0:

$$E\left\{\int_{0}^{t}e^{T}\left(t\right)e\left(t\right)\;ds\right\}\leq \frac{1}{\lambda_{0}}EV\left(0,\phi \left(t_{0}\right),r_{0},q_{0}\right),$$

based on Definition 4, which implies that error system (16) is globally, stochastically, asymptotically stable in the mean square sense. That means systems (1) and (2) get globally, stochastically, asymptotically synchronized in the mean square sense. The proof is completed. □

Notice that Theorem 1 only gives sufficient conditions for the synchronization of systems (1) and (2), and fails to solve the design problem of the controller (12). Therefore, the design method of the control gain *K* is constructed in Theorem 2.

**Theorem** **2.** *Suppose Assumption 1 holds. The FNNs (1) and (2) are globally, stochastically, asymptotically synchronized in the mean square sense, for the given scalars r, if there exist positive definite matrices $P,\Omega ,P_{1}^{im},P_{3}^{im},N_{1},N_{3}$, $R_{1}^{im},R_{2}^{im},M_{1}^{im},M_{2}^{im},L_{1}$, $L_{2},J_{1},J_{2},Q_{1},Q_{2}$; positive definite diagonal matrices $\Delta_{1},\Delta_{2}$; and matrices $P_{2}^{im},N_{2},S^{im}$, $T^{im},Y$; and positive scalars $\varepsilon ,\lambda_{1},\lambda_{2},$ such that the following LMIs for every i,m hold:*

(39) $$\begin{array}{c} \left[\begin{array}{ccccccc} {\tilde{\Pi }}_{1,1} & \Pi_{1,2} & \Pi_{1,3} & {\tilde{\Pi }}_{1,4} & \Pi_{1,5} & {\tilde{\Pi }}_{1,6} & {\tilde{\Pi }}_{1,7}\\ {}* & \Pi_{2,2} & \Pi_{2,3} & 0 & \Pi_{2,5} & 0 & 0\\ {}* & * & \Pi_{3,3} & 0 & 0 & 0 & {\tilde{\Pi }}_{3,7}\\ {}* & * & * & \Pi_{4,4} & 0 & {\tilde{\Pi }}_{4,6} & {\tilde{\Pi }}_{4,7}\\ {}* & * & * & * & \Pi_{5,5} & 0 & {\tilde{\Pi }}_{5,7}\\ {}* & * & * & * & * & {\tilde{\Pi }}_{6,6} & 0\\ {}* & * & * & * & * & * & {\tilde{\Pi }}_{7,7} \end{array}\right]<0, \end{array}$$

(40) $$\begin{array}{c} {\bar{R}}_{1}^{im}<L_{1},{\bar{R}}_{2}^{im}<L_{2},{\bar{M}}_{1}^{im}<J_{1},{\bar{M}}_{2}^{im}<J_{1}, \end{array}$$

(41) $$\begin{array}{c} \left[\begin{array}{cc} R_{2}^{im} & S^{im}\\ {}* & R_{2}^{im} \end{array}\right]\geq 0,\left[\begin{array}{cc} M_{2}^{im} & T^{im}\\ {}* & M_{2}^{im} \end{array}\right]\geq 0, \end{array}$$

(42) $$\begin{array}{c} \left[\begin{array}{cc} P_{1}^{im} & P_{2}^{im}\\ {}* & P_{3}^{im} \end{array}\right]>0,\left[\begin{array}{cc} N_{1} & N_{2}\\ {}* & N_{3} \end{array}\right]>0, \end{array}$$

*where*

$$\begin{array}{cc} {\tilde{\Pi }}_{1,1} & =\left[\begin{array}{cc} \Xi_{1,1} & {\tilde{\Xi }}_{1,2}\\ {}* & \Xi_{2,2} \end{array}\right],{\tilde{\Pi }}_{1,4}=\left[\begin{array}{ccc} b_{m}^{c}{\bar{b}}_{i}^{s}Y & {\bar{b}}_{m}^{c}P & b_{m}^{c}b_{i}^{s}Y\\ 0 & 0 & \lambda_{1}C^{T}G_{s}^{T}G_{s} \end{array}\right],{\tilde{\Pi }}_{1,6}=\left[\begin{array}{c} 0\\ b_{i}^{s}C^{T}Y^{T} \end{array}\right],\\ {\tilde{\Pi }}_{1,7} & =\Psi ⊗\left[\begin{array}{c} -A^{T}P\\ b_{i}^{s}b_{m}^{c}C^{T}Y^{T} \end{array}\right],{\tilde{\Pi }}_{3,7}=\Psi ⊗\left[\begin{array}{c} B^{T}P\\ D^{T}P \end{array}\right],{\tilde{\Pi }}_{4,6}=\left[\begin{array}{c} {\bar{b}}_{i}^{s}Y^{T}\\ 0\\ b_{i}^{s}Y^{T} \end{array}\right],\\ {\tilde{\Pi }}_{4,7} & =\Psi ⊗\left[\begin{array}{c} {\bar{b}}_{i}^{s}b_{m}^{c}Y^{T}\\ {\bar{b}}_{m}^{c}P\\ b_{i}^{s}b_{m}^{c}Y^{T} \end{array}\right],{\tilde{\Pi }}_{5,7}=\Psi ⊗\left[\begin{array}{c} 0\\ 0\\ G^{T}P \end{array}\right],{\tilde{\Pi }}_{6,6}=-2\alpha_{1}P+\alpha_{1}^{2}\lambda_{2}G_{c}^{T}G_{c},\\ {\tilde{\Pi }}_{7,7} & =diag\{-2\alpha_{2}P+\alpha_{2}^{2}R_{2}^{im},-2\alpha_{3}P+\alpha_{3}^{2}M_{2}^{im},-4h\alpha_{4}P+2h\alpha_{4}^{2}L_{2},-4h\alpha_{5}P+2h\alpha_{5}^{2}J_{2}\},\\ {\tilde{\Xi }}_{1,2} & =b_{m}^{c}b_{i}^{s}YC+R_{2}^{im}-S^{im}, \end{array}$$

*and the other parameters are the same as in Theorem 1, among them the feedback gain matrix is defined with $K=P^{-1}Y$.*

**Proof.**  For any scalar α>0, the following inequality holds:

$$\left(\alpha \Omega -P\right)\Omega^{-1}\left(\alpha \Omega -P\right)\geq 0.$$

Based on the inequality, it can be obtained that:

$$-P\Omega^{-1}P\leq -2\alpha P+\alpha^{2}\Omega .$$

By defining $\chi =diag\{{\overset{︷}{I,\ldots ,I}}^{13},P,P,P,P,P\},$ multipling (17) by χ on the left side and the right side, respectively, and replacing the term in $\Pi_{6,6}$ with $-2\alpha_{1}P+\alpha_{1}^{2}\lambda_{4}G_{c}^{T}G_{c}$, ${\tilde{\Pi }}_{6,6}$ can be obtained. In the same way, ${\tilde{\Pi }}_{7,7}$ replaces $\Pi_{7,7}.$ In addition, Y=KP is also replaced. Then linear matrix inequality (39) can be obtained. That completes the proof. □

## 4. Numerical Simulations

In this section, a simulation is presented to demonstrate the effectiveness of the proposed approach. Consider the FNNs which are described by Equation (1) and (2) with the following parameters:

$$A=\left[\begin{array}{cc} 1 & 0\\ 0 & 1 \end{array}\right],B=\left[\begin{array}{cc} 1.8 & -0.1\\ -2 & 0.4 \end{array}\right],D=\left[\begin{array}{cc} -1.7 & -0.6\\ 0.5 & -2.5 \end{array}\right],$$

$$E_{a}=\left[\begin{array}{cc} 0.01 & 0\\ 0 & 0.01 \end{array}\right],E_{b}=\left[\begin{array}{cc} 0.01 & 0\\ 0 & 0.01 \end{array}\right],E_{d}=\left[\begin{array}{cc} 0.01 & 0\\ 0 & 0.01 \end{array}\right],$$

$$G=\left[\begin{array}{cc} 0.01 & 0\\ 0 & 0.02 \end{array}\right],C=\left[\begin{array}{cc} 1 & 0\\ 0 & 1 \end{array}\right].$$

The nonlinear function was selected as $\hat{f}\left(x\right)=$ tanh(x), so it can be calculated that Φ=I. Due to the time-varying delay $\eta \left(t\right)=\frac{0.1e^{t}}{1+e^{t}}$, $\eta =0.1,\bar{\eta }=0.025$ can be obtained, respectively. The functions of deception signals are were chosen to be $g_{s}\left(x\right)=$ tanh$\left(x\right),g_{c}\left(x\right)=$ tanh(x); therefore, one can get $G_{s}=I,G_{c}=I$. In this numerical example, we set the sampling period to h=0.05, γ=0.98, the initial value of the adaptive event-triggered parameter $d_{0}$ to 0.8, the external input vector I(t) to 0, $\epsilon_{1}=0.1,\epsilon_{2}=0.1,\epsilon_{3}=0.1,\epsilon_{4}=0.1,\epsilon_{5}=0.1$. Additionally, the generators of Markov process $r_{t},q_{t}$ were

$$\pi_{ij}=\left[\begin{array}{cc} -0.4 & 0.4\\ 0.5 & -0.5 \end{array}\right],\rho_{ij}=\left[\begin{array}{cc} -0.4 & 0.4\\ 0.65 & -0.65 \end{array}\right].$$

Based on the proposed method, by solving the LMIs in Theorem 2, one can obtain the desired controller gain and the adaptive event-triggered weighting matrix as follows:

(43) $$\begin{array}{c} K=\left[\begin{array}{cc} -0.0178 & 0.0026\\ -0.0021 & -0.0270 \end{array}\right],\Omega =\left[\begin{array}{cc} 0.0007 & 0.0007\\ 0.0007 & 0.0011 \end{array}\right]. \end{array}$$

**Figure 2:**
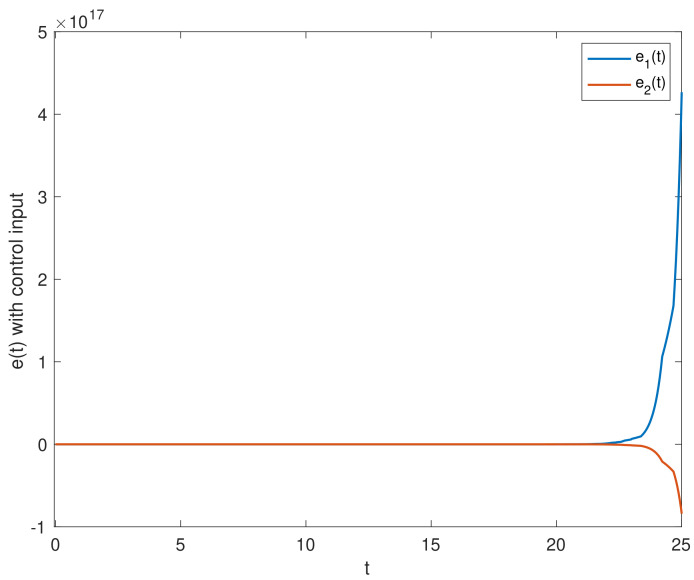
Synchronization error $e_{i}\left(t\right)\left(i=1,2\right)$ without control input.

**Figure 3:**
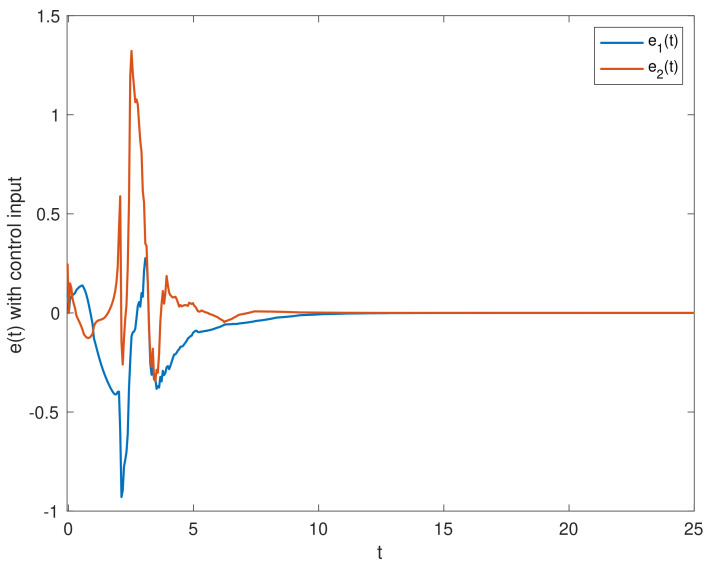
Synchronization error $e_{i}\left(t\right)\left(i=1,2\right)$ with control input $u_{i}\left(t\right)\left(i=1,2\right)$.

**Figure 4:**
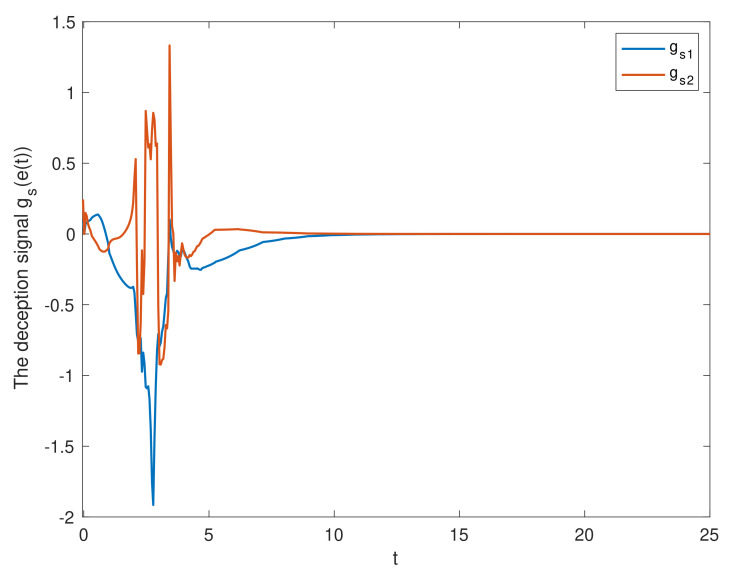
The state of the deception signal in the S-C channel.

**Figure 5:**
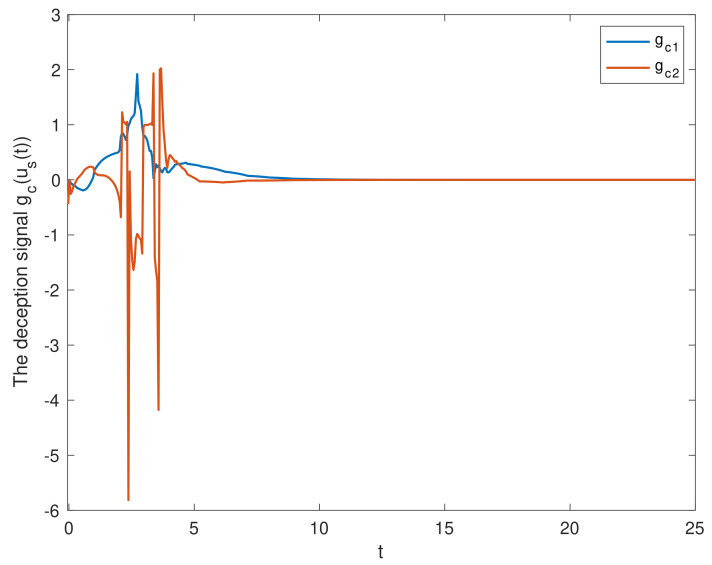
The state of the deception signal in the C-A channel.

**Figure 6:**
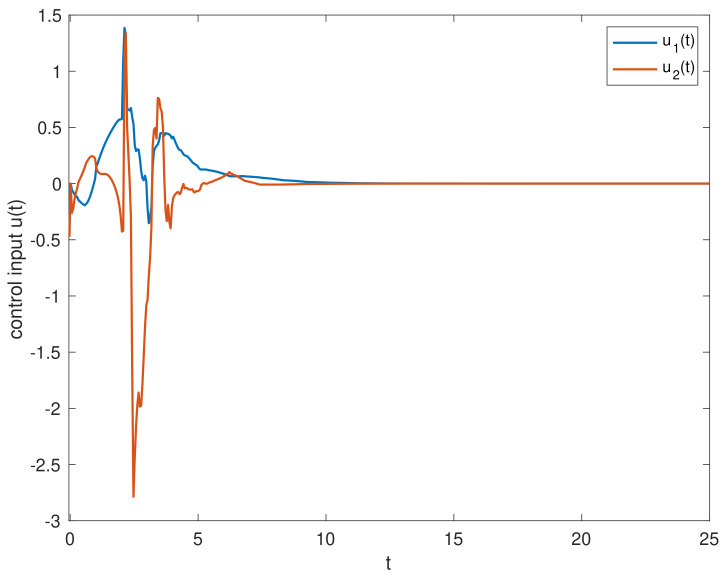
The trajectories of control input $u_{i}\left(t\right)\left(i=1,2\right)$.

**Figure 7:**
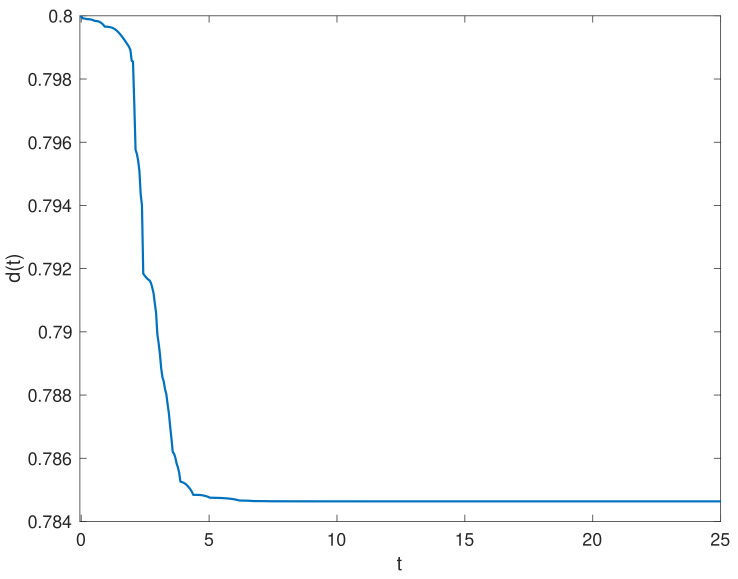
The trajectory of event-triggered parameter d(t).

We chose the initial values $\phi_{1}\left(t_{0}\right)=\left(0.5;-0.1\right)$, $\phi_{2}\left(t_{0}\right)=\left(0.1;0.2\right)$. Figure 2 shows the state trajectories of synchronization errors without control input. As can be seen from Figure 2, if there is no control input, the error system itself is unstable, which means that the systems cannot be synchronized. Using the feedback controller (12), the simulation results were obtained, as shown in Figure 3, Figure 4, Figure 5, Figure 6 and Figure 7. Figure 3 shows the state trajectories of synchronization errors with control input, and one can see that synchronization errors finally converged to zero under the designed control protocol, which shows that the systems can achieve synchronization. Figure 4 and Figure 5 depict the states of double deception attacks, whose states caused the oscillations of the synchronization error and the control input. Figure 6 depicts the trajectories of control input, from which one can see that the control input gradually tended to 0; that is, when the system achieves synchronization, external control is no longer required. Figure 7 shows the evolution of adaptive threshold coefficient d(t) in AETS. From the adaptive law (6), the adaptive threshold coefficient can be timely adjusted according to the synchronization error. Therefore, when the error system is stable, that is, when synchronization is achieved, the parameter will no longer be adjusted and will tend toward a constant. From the above simulation results, it can easily be seen that the proposed synchronization problem in this paper was effectively solved.

## 5. Discussion and Conclusions

The adaptive event-triggered synchronization problem of uncertain FNNs with double deception attacks and time-varying delay has been investigated in this paper. Noteworthy is that, regarding fractional order systems receiving deception attacks using traditional event-triggered methods given in the literature [22], we believe that the literature has not been comprehensive enough. Not only the traditional ETS technology, but also the attack phenomena were governed by Bernoulli processes, and attacks only occurred in the C-A channel. Thus, in this study, the AETS was adopted to determine the signals the needed to be transmitted. The deception attacks in communication channels from the sensor to controller and from controller to actuator are governed by two independent Markov processes. Considering the AETS, double deception attacks, and parameter uncertainties, a time-varying closed-loop fractional order synchronization error system was constructed. Sufficient conditions were formulated to guarantee the considered system is stochastically stable by employing the Lyapunov–Krasovskii functional method. Finally, a numerical example was presented to verify its effectiveness and the feasibility of the proposed method. Thereby, we showed that our approach is more meaningful and comprehensive. It should be mentioned that besides deception attacks, denial of service (DoS) attacks is another interesting issue for FNNs and deserves further exploration. In addition, solving the problem of multiple communication channels for FNNs will be part of our future research efforts.

## Data Availability

Not applicable.

## Abbreviations

The following abbreviations are used in this paper:

FNNs	Fractional order neural networks
ETS	Event-triggered scheme
TTS	Time-triggered scheme
AETS	Adaptive event-triggered scheme
S-C Channel	Sensor to controller channel
C-A Channel	Controller to actuator channel
